# C-Peptide and Its C-Terminal Fragments Improve Erythrocyte Deformability in Type 1 Diabetes Patients

**DOI:** 10.1155/2008/730594

**Published:** 2008-05-06

**Authors:** Thomas Hach, Thomas Forst, Thomas Kunt, Karin Ekberg, Andreas Pfützner, John Wahren

**Affiliations:** ^1^Department of Internal Medicine, University of Mainz, 55101 Mainz, Germany; ^2^McKinsey & Company, Inc., Am Sandtorkai 77, 20457 Hamburg, Germany; ^3^Institute for Clinical Research and Development, 55116 Mainz, Germany; ^4^Department of Internal Medicine, Zayed Military Hospital, P.O. Box 3740, Abu Dhabi, United Arab Emirates; ^5^Department of Molecular Medicine and Surgery, Section of Clinical Physiology, Karolinska Institute, 17177 Stockholm, Sweden

## Abstract

*Aims/hypothesis*. Data now indicate that proinsulin C-peptide exerts important physiological effects and shows the characteristics of an endogenous peptide hormone. This study aimed to investigate the influence of C-peptide and fragments thereof on erythrocyte deformability and to elucidate the relevant signal transduction pathway. *Methods*. Blood samples from 23 patients with type 1 diabetes and 15 matched healthy controls were incubated with 6.6 nM of either human C-peptide, C-terminal hexapeptide, C-terminal pentapeptide, a middle fragment comprising residues 11–19 of C-peptide, or randomly scrambled C-peptide. Furthermore, red blood cells from 7 patients were incubated with C-peptide, penta- and hexapeptides with/without addition of ouabain, EDTA, or pertussis toxin. Erythrocyte deformability was measured using a laser diffractoscope in the shear stress range 0.3–60 Pa. *Results*. Erythrocyte deformability was impaired by 18–25% in type 1 diabetic patients compared to matched controls in the physiological shear stress range 0.6–12 Pa (*P* < .01–.001). C-peptide, penta- and hexapeptide all significantly improved the impaired erythrocyte deformability of type 1 diabetic patients, while the middle fragment and scrambled C-peptide had no detectable effect. Treatment of erythrocytes with ouabain or EDTA completely abolished the C-peptide, penta- and hexapeptide effects. Pertussis toxin in itself significantly increased erythrocyte deformability. *Conclusion/interpretation*. C-peptide and its C-terminal fragments are equally effective in improving erythrocyte deformability in type 1 diabetes. The C-terminal residues of C-peptide are causally involved in this effect. The signal transduction pathway is Ca^2+^-dependent and involves activation of red blood cell Na^+^, K^+^-ATPase.

## 1. INTRODUCTION

During the past decade, several studies have provided evidence that C-peptide is a biologically active endogenous peptide. It binds specifically in nanomolar concentration to cell membranes [1], possibly to a G-protein-coupled receptor, resulting in internalisation of the peptide [2] and activation of Ca^2+^- and MAPK-dependent signalling pathways [3–6]. Cellular end-effects include stimulation and induction of both Na^+^, K^+^-ATPase, and eNOS as well as activation of a series of transcriptional fators [3, 7–10]. Studies in patients with type 1 diabetes who lack endogenous C-peptide production show that administration of C-peptide in replacement doses results in increased regional blood flow in several tissues [11–13] and amelioration of diabetes-induced functional and structural abnormalities of the peripheral nerves and the kidneys [14–16]. Not only the full-length native C-peptide but also its C-terminal pentapeptide fragment is reported to exert physiological effects [1, 4, 6, 17, 18].

It is well established that diabetes is associated with reduced deformability of red blood cells [19–21]. This abnormality is of clinical significance in that it compromises the ability of red blood cells to pass through capillaries, reduces tissue perfusion, and impairs tissue oxygen supply [4, 22]. The mechanism underlying the decreased red cell compliance in diabetes is not well understood, but it has been shown that C-peptide is capable of restoring red cell deformability to normal levels, most likely via stimulation of red cell Na^+^, K^+^-ATPase [23]. The extent to which C-peptide fragments will do the same has not been determined. Consequently, the aim of this study was to examine the influence of C-peptide and its C-terminal fragments, as well as middle segments on the abnormal deformability of red blood cells from type 1 diabetes patients. In addition, aspects of a possible signal transduction pathway were evaluated.

## 2. SUBJECTS, MATERIALS, AND METHODS

### 2.1. Subjects

 Blood samples were taken from 23 type 1 diabetes patients and 15 matched healthy controls (Table 1). Inclusion criteria were C-peptide serum level < 0.2 nmol/L, diabetes duration > 2 years, serum creatinine < 2 mg/dL, and no clinically relevant diabetic long-term complications. Hematocrit values were in the normal range for all patients and controls.

### 2.2. Materials and methods

 Laser diffractoscopy was performed using a Rheodyn SSD shear stress diffractometer (Myrenne GmbH, Roetgen, Germany). The method of laser diffractoscopy has been described in detail previously [21, 23]. In summary, a He-Ne-Laser detects deformation of erythrocytes between two parallel glass discs, one of which rotates resulting in defined shear stresses as per the following equation:

(1) $$\tau =\frac{2\pi r\cdot \eta }{t\cdot h},$$

where *τ* = shear stress, *r* = laser beam distance from rotation center, *η* = viscosity, *h* = height of gap between discs, and *t* = time.

Adjusting for equipment-specific values (*r* = 25 mm, *h* = 0.5 mm, and *η* = 24 mPa), the equation is condensed to

(2) τ=7.536Pa⋅rpm,

where rpm = revolutions per minute.

The applied shear stress range is electronically regulated and includes 8 levels (0.3 Pa; 0.6 Pa; 1.2 Pa; 3 Pa; 6 Pa; 12 Pa; 30 Pa; 60 Pa).

The erythrocyte deformability measurement detects scattered-light intensities along orthogonal axes (A, B) of red blood cells within the laser diffraction light cone. The erythrocyte elongation index (EI) is calculated by the following equation:

(3) $$\text{EI}\left(\%\right)=(\frac{\text{A}-\text{B}}{\text{A}+\text{B}})\cdot 100.$$

All experiments were carried out at 23°C, which was thermostatically regulated. Blood samples were collected using ammonium-heparinated vials and subsequently distributed to dextran medium (30 *μ*L blood per 2 mL dextran). Stability tests were performed to exclude significant effects of storage time and/or serum glucose concentration on erythrocyte deformability measured by laser diffractoscopy (data not shown). All measurements of erythrocyte deformability were carried out before incubation with active ingredients as well as at 1, 30, and 60 minutes after incubation

**C-peptide and C-peptide fragments**

Human C-peptide and all C-peptide fragments were provided by Creative Peptides (Stockholm, Sweden) at a purity of >98%. C-peptide and its fragments were dissolved in phosphate buffer and incubations were made at 6.6 nmol/L. The incubation and mixing of probes were done smoothly to avoid external shear stress. The fragments used in this study were C-terminal hexapeptide, C-terminal pentapeptide, a middle fragment including residues 11–19, and randomly scrambled C-peptide; their amino acid sequences are presented in Table 2.

**Ouabain**

Ouabain was obtained from Sigma-Aldrich (St. Louis, Mo, USA). Treatment with ouabain was titrated to achieve a concentration of 1 *μ*mol/L. Incubations with and without C-peptide or fragments lasted 30 minutes at 23°C.

**EDTA**

Incubations with EDTA (1.6 mg/mL) with and without C-peptide or fragments lasted 30 minutes at 23°C. Treatment with EDTA alone did not alter erythrocyte deformability (data not shown).

**Pertussis toxin**

Pertussis toxin was obtained from Sigma-Aldrich. Treatment with pertussis toxin (1 *μ*g/mL) was conducted for 60 minutes at 37°C.

**Statistical analysis**

Results are expressed as the means ± SD. Gaussian distribution was checked by the Kolmogoroff-Smirnoff test. Statistical analysis were done by two-site ANOVA and Student's *t* test. A *P*-value of less than .05 was regarded as statistically significant.

## 3. RESULTS

**Figure 1:**
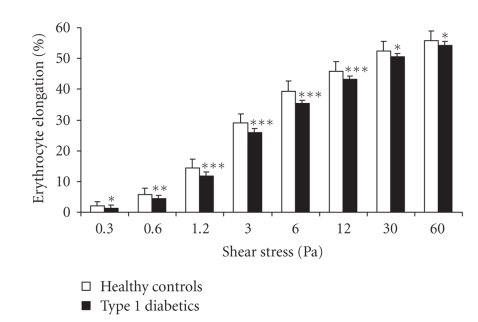
Erythrocyte elongation index (EI) (%) ± SD for erythrocytes from healthy controls (open columns) and type 1 diabetes patients (filled columns) at different levels of shear stress. *P < .05. **P < .01. ***P < .001 versus controls.

Erythrocyte deformability was significantly decreased in type 1 diabetes patients compared to healthy controls over the full range of shear stress tested (Figure 1). In the physiological shear stress range (≤12 Pa), the difference between diabetic patients and healthy controls amounted to 18–25% (*P* < .01–.001) Incubation of the red cells from type 1 diabetes patients with C-peptide completely normalized the erythrocyte defortmability at all tested shear stress levels (Table 3). Incubation with the penta- and hexapeptides also resulted in significant improvements in erythrocyte deformability over the range of shear stress tested; the responses to C-peptide and the C-terminal peptides were similar and there were no statistically significant differences between these three treatment groups. In contrast, the middle fragment exerted no significant effect on the diabetes-induced abnormal erythrocyte deformability. Likewise and as expected, scrambled C-peptide with a random amino acid sequence had no beneficial effect on erythrocyte deformability.

**Figure 2:**
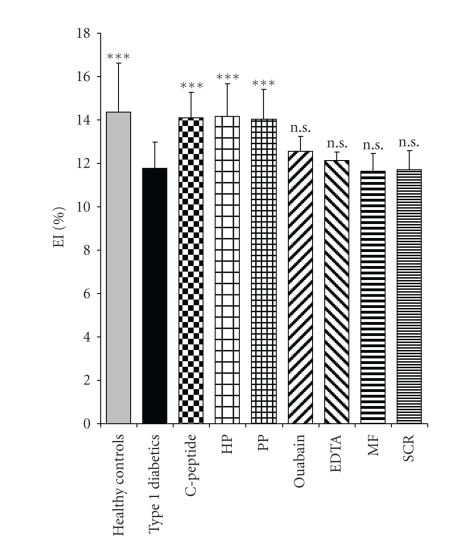
Erythrocyte elongation index **(**EI) (%) ± SD for erythrocytes from healthy controls and type 1 diabetes patients. The latter cells were incubated with C-peptide, hexapeptide (HP), and pentapeptide (PP) from the C-terminal region of C-peptide as well as a middle fragment (MF) and scrambled peptide (SCR, control). Results for combined incubation with PP and oubain or EDTA are also shown. Data are obtained at the shear stress level 1.2 Pa. ***P < .001 versus untreated erythrocytes from type 1 diabetes patients.

Pretreatment of erythrocytes from patients with type 1 diabetes with ouabain or EDTA completely abolished the C-peptide-, penta- and hexapeptide-induced improvements in deformability as shown in Figure 2 for the pentapeptide at the shear stress 1.2 Pa. Similar results were obtained for all levels of shear stress. Pretreatment of erythrocytes with pertussis toxin in itself increased erythrocyte deformability significantly (*P* < .05) in the shear stress range of 0.6–12 Pa. Therefore, the possible influence of G-protein inactivation on the effects mediated by C-peptide and its fragments could not be evaluated.

## 4. DISCUSSION

The present results confirm and extend previous observations indicating that red blood cell deformability is compromised in diabetes [19–21] and that this abnormality can be corrected by C-peptide [23]. Thus, using whole blood samples and laser diffractoscopy, a method with high reproducibility (CV < 1%), erythrocyte deformability measured in blood samples from type 1 diabetes patients was found to be reduced by 18–25% over the physiological shear stress range (0.6–12 Pa), in keeping with previous results using the same methodology [23]. Exposing blood cells from the diabetic patients to 6.6 nM, C-peptide was found to fully normalize membrane deformability. We now show that not only the native, full length C-peptide but also its C-terminal penta- and hexapeptides possess this capability. In contrast, a middle segment comprising residues 11–19 of C-peptide had no effect. The specificity of the observed effects is attested by the finding that scrambled C-peptide, a control peptide with its residues assembled in random order, had no detectable effect.

The current results for the pentapeptide are in line with previous reports that it competes with C-peptide for cellular binding [1], that it elicits an increase in intracellular Ca^2+^-concentration [4], activates PKC isoforms, causes phosphorylation of ERK1/2 and JNK, and stimulates Na^+^, K^+^-ATPase activity [6, 17, 24]. Studies evaluating the pentapeptide's cellular binding/competition characteristics, its stimulatory effect on intracellular Ca^2+^-concentrations and ERK1/2 phosphorylation demonstrate that it is the N-terminal Glu-residue of the pentapeptide (Glu 27 of C-peptide) that is of primary importance for its bioactivity; alanine substitution of the other four residues has little or no effect on the pentapeptide's bioactivity [4, 25, 26]. Its bioactivity has been found to be similar to that of the native peptide both in the above in vitro studies and in invivo experiments evaluating its stimulatory effect on whole-body glucose utilization [18] and its inhibition of diabetes-induced glomerular hyperfiltration [27] in streptozotocin-diabetic rats.

The C-terminal hexapeptide was found to be equally potent as the pentapeptide and the native C-peptide with regard to its amelioration of diabetes-induced abnormal red blood cell deformability (Figure 2). Only little information is available with regard to the physiology of the hexapeptide but the current findings are in line with previous observations that it is capable of stimulating Na^+^, K^+^-ATPase in renal tubular segments with potency similar to that of the pentapeptide [17]. In contrast to the findings for the two C-terminal segments, there was no indication that the middle fragment (residues 11–19) exerted a measurable effect on diabetes-induced abnormal red cell deformability. Variable results have been reported previously for middle fragments; mild stimulation of Na^+^, K^+^-ATPase in renal tubule segments [17] but no effect on glucose utilization under in vivo conditions [18] have been observed.

Exposing red blood cells from patients with type 1 diabetes to either oubain or EDTA resulted in complete abrogation of the normalizing effect by the penta- and hexapeptides or C-peptide on erythrocyte deformability. Evaluation of a G-protein involvement in the signalling pathway could not be carried out since exposure of the erythrocytes to pertussis toxin in itself resulted in alteration of red cell deformability in contrast to previous findings for rabbit erythrocytes [28]. Nevertheless, the results indicate that the beneficial effects of the peptides are mediated via a Ca^2+^-dependent stimulation of erythrocyte Na^+^, K^+^-ATPase, in analogy with the previously established signal transduction pathway for C-peptide and Na^+^, K^+^-ATPase in human renal tubular cells [24], even though the EDTA results may suggest that other metal ions also are of importance for the signal transduction [29].

Exposure of erythrocytes to C-peptide and its C-terminal fragments is thus likely to result in augmented generation and release of ATP, as has been observed directly in the case of C-peptide and both normal and diabetic red blood cells [29]. ATP is a recognized stimulus of nitric oxide synthase of platelets and erythrocytes [30, 31]. Nitric oxide, besides being a potent vasodilator, is also effective in eliciting improved red blood cell deformability as a result of direct action of nitric oxide on the erythrocyte membrane [32, 33]. In addition, C-peptide-related improvement of red cell deformability may be mediated not via ATP but by direct stimulation of red cell nitric oxide synthase. The latter is functionally similar to that of endothelial cells [31], and C-peptide and most likely also its C-terminal fragments are known to stimulate and cause induction of endothelial nitric oxide synthase [8, 10]. The above mechanisms are likely to contribute to the maintenance of normal erythrocyte membrane plasticity and the extent to which they are modified in diabetes is unknown. However, irrespective of the exact mechanism involved, the present findings demonstrate that both C-peptide and its C-terminal fragments are capable of effectively normalizing the diabetes-induced reduction in red blood cell deformability. It is probable that this effect contributed importantly to the observed improvement in regional blood flow of skin, muscle, myocardium, and peripheral nerve in animals and type 1 diabetes patients, following administration of C-peptide to physiological concentrations [11–13, 34]. Furthermore, the beneficial clinical effects of C-peptide on early-stage neuropathy [14] and nephropathy [15] in type 1 diabetes may in part be related to correction of the abnormal red cell deformability and subsequently improved microcirculation. Further clinical trials are warranted document and define the role of C-peptide and its C-terminal fragments in the treatment and/or prevention of microvascular complications of type 1 diabetes.

## Figures and Tables

**Table 1:** Clinical characteristics of study subjects.

	Healthy controls *n* = 15	Type 1 diabetes patients *n* = 23
Sex (male/female)	8/7	13/10
Age (years)	34 ± 4	36 ± 3
Height (m)	1.76 ± 0.02	1.75 ± 0.02
Weight (kg)	78.0 ± 2.8	77.5 ± 2.6
Diabetes duration (years)	0 ± 0	24 ± 3
Serum glucose (mmol/L)	4.94 ± 0.22	7.44 ± 0.67
HbA1c (%)	5.7 ± 0.3	7.3 ± 0.2
Serum C-peptide (nmol/L)	0.77 ± 0.09	0.01 ± 0.003

**Table 2:** Amino acid structure of C-peptide and C-peptide fragments used in this study.

	Amino acid structure	Position
C-Peptide	EAEDLQVGQVELGGGPGAGSLQPLALEGSLQ	(1–31)
MF	ELGGGPGAG	(11–19)
HP	LEGSLQ	(26–31)
PP	EGSLQ	(27–31)

**Table 3:** Erythrocyte elongation index (EI) (%) in the shear stress range 0.6–12 Pa for different treatment groups, *n* = 23.

Shear stress					
	0.6 Pa	1.2 Pa	3 Pa	6 Pa	12 Pa
Healthy controls	5.87 ± 0.72*	14.37 ± 0.61*	29.08 ± 0.79*	39.19 ± 1.02*	45.93 ± 0.85*
Diabetes patients	4.38 ± 0.63	11.77 ± 0.49	26.01 ± 0.60	35.48 ± 0.84	43.34 ± 0.92
C-peptide	5.40 ± 0.82*	14.11 ± 0.85*	28.34 ± 1.58*	39.07 ± 1.46*	45.82 ± 1.43*
HP	5.77 ± 0.81*	14.18 ± 1.17*	28.61 ± 1.37*	38.49 ± 1.62*	45.82 ± 1.81*
PP	6.12 ± 0.93*	14.02 ± 1.37*	28.23 ± 1.46*	38.85 ± 1.54*	45.20 ± 1.74*
MF	4.42 ± 0.89	11.39 ± 1.07	25.82 ± 1.24	35.79 ± 0.98	43.39 ± 1.17
SCR	4.10 ± 0.91	11.25 ± 1.07	25.58 ± 1.07	35.64 ± 0.91	43.48 ± 1.00

*P < .01 versus type 1 diabetes patients.
